# Identification of an intestine-specific promoter and inducible expression of bacterial α-galactosidase in mammalian cells by a *lac* operon system

**DOI:** 10.1186/2049-1891-3-32

**Published:** 2012-10-30

**Authors:** Zhai Ya-Feng, Shu Gang, Zhu Xiao-Tong, Zhang Zhi-Qi, Lin Xia-Jing, Wang Song-Bo, Wang Li-Na, Zhang Yong-Liang, Jiang Qing-Yan

**Affiliations:** 1 College of Animal Science, South China Agricultural University, Guangzhou 510642, China

**Keywords:** α-galactosidase, Inducible expression, Intestine-specific promoters, *Lac* operon

## Abstract

**Background:**

α-galactosidase has been widely used in animal husbandry to reduce anti-nutritional factors (such as α-galactoside) in feed. Intestine-specific and substrate inducible expression of α-galactosidase would be highly beneficial for transgenic animal production.

**Methods:**

To achieve the intestine-specific and substrate inducible expression of α-galactosidase, we first identified intestine-specific promoters by comparing the transcriptional activity and tissue specificity of four intestine-specific promoters from human intestinal fatty acid binding protein, rat intestinal fatty acid binding protein, human mucin-2 and human lysozyme. We made two chimeric constructs combining the promoter and enhancer of human mucin-2, rat intestinal trefoil factor and human sucrase-isomaltase. Then a modified *lac* operon system was constructed to investigate the induction of α-galactosidase expression and enzyme activity by isopropyl β-D-1-thiogalactopyranoside (IPTG) and an α-galactosidase substrate, α-lactose.

We declared that the research carried out on human (Zhai Yafeng) was in compliance with the Helsinki Declaration, and experimental research on animals also followed internationally recognized guidelines.

**Results:**

The activity of the human mucin-2 promoter was about 2 to 3 times higher than that of other intestine-specific promoters. In the *lac* operon system, the repressor significantly decreased (*P* < 0.05) luciferase activity by approximately 6.5-fold and reduced the percentage of cells expressing green fluorescent protein (GFP) by approximately 2-fold. In addition, the expression level of α-galactosidase mRNA was decreased by 6-fold and α-galactosidase activity was reduced by 8-fold. In line with our expectations, IPTG and α-lactose supplementation reversed (*P* < 0.05) the inhibition and produced a 5-fold increase of luciferase activity, an 11-fold enhancement in the percentage of cells with GFP expression and an increase in α-galactosidase mRNA abundance (by about 5-fold) and α-galactosidase activity (by about 7-fold).

**Conclusions:**

We have successfully constructed a high specificity inducible *lac* operon system in an intestine-derived cell line, which could be of great value for gene therapy applications and transgenic animal production.

## Introduction

α-galactosidase, an exo-glycosidase enzyme that hydrolyzes Gal glycosidic bonds, has been widely used in both medical research and animal husbandry. For example, the α-galactosidase gene has been investigated for the gene therapy of Fabry disease, which is caused by a defect in α-galactosidase activity [1]. In addition, α-galactosidase has been used as a tool for changing blood type B to O because of its ability to diminish the cell membrane antigen [2]. In animal husbandry, anti-nutritional factors (like α-galactoside in soybean meal) can cause flatulence and even permeability diarrhea in monogastric animals [3], while α-galactosidase supplementation in diets can not only improve the utilization of oligosaccharides and nutrient absorption in the intestinal tract but can also improve growth performance [4-6].

Human α-galactosidase has been used in a transgenic mouse model for Fabry disease [1,7]; however, the cytomegalovirus (CMV) promoter used in these studies demonstrated poor and nonspecific expression of α-galactosidase in almost all tissues. Therefore, abundant expression during the embryonic stage might cause embryos to be aborted or stillborn [8].

To achieve the specific expression of an exogenous gene in the gastrointestinal tract, several promoters have been used for cell-specific gene delivery, such as the human intestinal fatty acid binding protein promoter (HIFABP) [9], the rat intestinal fatty acid binding protein promoter (RIFABP) [10], the human mucin-2 promoter (HMUC2) [11], the human lysozyme promoter (HLY) [12], the human sucrase-isomaltase enhancer (HSI) [13] and the rat intestinal trefoil factor (RITF) [14]. Although the activities of these intestine-specific promoters are known, the regulatory element that is most appropriate for directing α-galactosidase expression in intestinal cells still required identification.

Some binary systems, such as the *lac* operon system [15], the tetR-based system [16], the GAL4-based system [17] and the Cre/loxP recombination system [18], have been used to turn gene expression on and off transiently or permanently. Bacterial *lac* operon-regulated gene expression in mammalian cells was first demonstrated by Hu and Davidson [19]. Cronin et al. [15] also showed that the *lac* operon system was functional in the mouse and could provide tight and reversible gene expression. The *lac* operon induction system might be the best option for inducible α-galactosidase expression because α-galactosidase hydrolysis of oligosaccharides or milk can produce α-lactose, which binds to the *lac* repressor and facilitates positive feedback of α-galactosidase expression.

To achieve intestine-specific and substrate inducible expression of α-galactosidase, we first compared the activity and cell specificity of several regulatory elements associated with intestinal gene expression. Then an α-galactosidase inducible expression vector was constructed based on the *lac* operon system. Luciferase activity and α-galactosidase activity were also investigated in response to isopropyl-β-D-thiogalactoside (IPTG) and α-lactose to assess the construct’s ability to regulate induction of target gene expression.

## Materials and methods

### Plasmid construction

**Figure 1:**
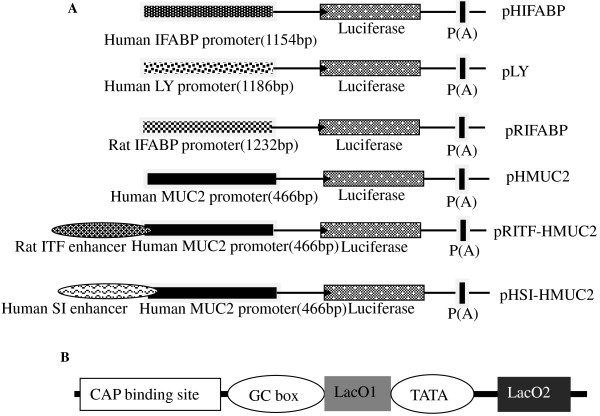
**Schematic structure of the intestine**-**specific promoters and operator.** (**A**) Promoter/enhancer fusion constructs. Four intestine-specific promoters were cloned into a pGL3-Basic vector to drive luciferase expression. In addition, the human mucin-2 promoter (HMUC2) sequence was fused to the human sucrase–isomaltase (HSI) and rat intestinal trefoil factor (RITF) regions to drive luciferase expression. (**B**) Schematic structure of the synthetic 116 bp operator, which consists of the two important components: LacO1 and LacO2.

Three human intestine-specific promoters, HIFABP, HMUC2 and HLY, and the HSI enhancer were amplified by PCR using human genomic DNA template. The RIFABP promoter was amplified by PCR using rat genomic intestinal DNA template. The primers used are shown in Table 1. The 59 bp RITF enhancer was chemically synthesized. Six plasmids with four different promoters and two different enhancers were constructed using the pGL3-Basic plasmid backbone (Promega, Shang Hai, China) (Figure 1A).

**Table 1:** Primers and regulatory element sequences used in this paper

**Name**^**1**^	**GenBank**	**Primer sequenc (5’-3’)**	**Product** (**bp**)
HMUC2	U67167	S:5’-CTAGCTAGCTCCTCCCAGCGTAACGTGAGC-3’	466
		A:5’-GAAGATCTCTAGTGGCAGCCCCATGGTG-3’	
HIFABP	NG_011444	S:5’-CCGACGCGTGTTAGATTTATCTTCCCTTGACC-3’	1154
		A:5’-CCGCTCGAGTACCTTCCAAGTGCTGTCAAAC-3’	
RIFABP	NW_047627	S:5’-CGACGCGTCATGCTGAATTCCTTAATTTGC-3’	1232
		A:5’-CCGCTCGAGCAGCTGTGTGTGCCTCTAGG-3’	
HLY	NM_000239	S:5’-CTAGCTAGCCTGTCCTCTTAGGCAGATACAGA-3’	1186
		A:5’- GAAGATCTAGAGCCTTCATGTTGACTGCTA-3’	
HSI	X85797	S: 5’- GGGGTACCCAATGAGTGCTATCTGTGGT-3’	230
		A: 5’-CGACGCGTAAGGAAAGCTGCTTAGGTA-3’	
LacI	V00294	S: 5'-CCCAAGCTTCCGGAAGAGAGTCAATTCAG-3'	1143
		A:5'-GCTCTAGAAGTTTCGAAGGAGAAGAAGAATCCCTGCCCGCTTTCCAGTC'-3’	
GFP	AY268072.1	S: 5'-CCAAGCTTTTGTTTCGTTTTCTGTTCTGCG-3'	763
		A: 5'-GCTCTAGAGCAGCGTATCCACATAGCGTAA-3'	
α-Gal	DQ344486	S: 5’-CATGCCATGGGCGGATGGTACTCTCTTTG-3’	2153
		A: 5’-GCTCTAGACTATTGCTTTTCCAACATCA-3’	
RITF enhancer		5’-CGGGGTACCTGTTTTCCTCCCTAACCCTCTCCCCTCCCCCTCGGACTCCCACGCGTCGC-3’	59

^1^HMUC2 = human mucin-2 promoter; HIFABP = human intestinal fatty acid binding protein promoter; RIFABP = rat intestinal fatty acid binding protein promoter; HLY = human lysozyme promoter; HSI= human sucrase-isomaltase enhancer; LacI = *lac* operon repressor; GFP = green fluorescent protein; α-Gal = α-galactosidase; RITF = rat intestinal trefoil factor.

To construct the *lac* operon, the repressor gene (*LacI*) was isolated from wild-type *Escherichia coli*, and a nuclear localization signal (NLS) was added during PCR amplification [20,21]. *LacI* was then cloned in a pGL3-control vector (Promega, China) by replacing the firefly luciferase (*luc*) gene. The 116 bp operator (*LacO*) (Figure 1B) was chemically synthesized and cloned in a pGL3-Basic vector [22,23]. The reporter plasmid, pBGFP, was constructed by replacing the *luc* gene with green fluorescent protein (GFP) (from pshRNA-copGFP Lentivector) as a negative control.

α-galactosidase (α-Gal) was obtained from a pPIC9-Agil vector (a gift from Prof. Yao, Feed Research Institute Chinese Academy of Agricultural Sciences, China) and then cloned into a pHMLacO vector by replacing the *luc* gene. The plasmid , pHMLacO which luc gene was replaced by α-Gal without the HMUC2 promoter was used as a negative control.

### Cell culture and DNA transfection

Cells, including IPEC-1 (pig\jejunum), HeLa (human/cervical), HepG2 (human\hepatic), NIH-3T3 (mouse/embryonic fibroblast), 293T (human/kidney) and CHO (hamster/ovary) were cultured in Dulbecco’s modified Eagle’s medium, and SW480 (human/colon) cells were cultured in Leibovitz’s L-15 medium (Gibco, China) with 10% fetal bovine serum, penicillin G (100 U/mL), streptomycin (100 μg/mL), and 2 mmol/L L-glutamine at 37°C in 5% CO_2_/air. The IPEC-1 cell line was kindly provided by Dr. Kong (Institute of Subtropical Agriculture, the Chinese Academy of Sciences) and other cell lines were obtained from the cell bank of Zhongshan Medical College.

Transient transfections were performed using GenEscort™ III (Wisegen, China) with approximately 1.5x10^5^ cells/well in 24-well dishes and 2 μg of each DNA. Plasmids were purified with the E.Z.N.A^TM^ EndoFee Plasmid Kit (Omega, China). In all cases, 200 ng of a plasmid containing the Renilla luciferase gene driven by the TK promoter (pRL-TK vector, Promega, China) was included in the assay to monitor transfection efficiency. After 48 h, cells were measured for luciferase activity. For induction experiments, both IPTG (Sigma, China) and α-lactose (Sigma, China) were prepared as 100 mmol/L stock solutions in Dulbecco’s modified phosphate-buffered saline without Ca^2+^ and Mg^2+^, pH 7.4 (PBS-).

For the transfection of a single plasmid, 4 μg of plasmid DNA was mixed with 10 μL of GenEscort™ III in PBS(−), and made up to a total of 200 μL with Opti-MEM (Gibco, China). For co-transfection, two plasmids (3 μg each) were mixed with 14 μL of GenEscort™ III in Opti-MEM to a total of 200 μL. These DNA/liposome complexes were added to approximately 5x10^5^ cells/well in 6-well dishes and incubated for 48 h at 37°C. The DNA/liposome complexes were added to SW480 cells cultured in 25 cm^2^ cell culture bottles and incubated for 96 h. For RNA extraction and α-galactosidase activity assays, medium was replaced by PBS prior to processing, while for induction experiments, medium was replaced with fresh media containing 2.5 mmol/L IPTG or 5 mmol/L α-lactose. The cells were induced for 12 h before being observed for fluorescence and harvesting for FACS.

### Fluorescence detection

Cells were observed using a Leica DMI 4000B fluorescence microscope (Leica) with DM505 filters (BP460-490 and BA510IF) for GFP monitoring. Microphotographs were taken using a digital camera (FUJIX HC-300/OL, FujiFilm) attached to the fluorescence microscope.

### Fluorescence Activated Cell Sorting (FACS) analysis

Forty-eight hours after transfection, cells were washed twice with PBS(−) and dissociated with trypsin/EDTA. After centrifugation at 1,000 rpm for 5 min at 4°C, cells were resuspended at 1x10^6^ cells/mL in 1x Assay Buffer (KeyGEN, China) and stored on ice for a maximum of 1 h before analysis. Then 5 μL 7-AAD (KeyGEN, China) was added immediately prior to flow cytometry. Acquisition was performed on a FACSCalibur system (BD Bioscience), and samples were analyzed using Cell Quest Pro software (BD Bioscience). In each experiment, 10,000 counts were evaluated. Cells exhibiting 7-AAD uptake were considered dead and excluded from the analysis of GFP-positive cells by gating on 7-AAD negative cells.

### Luciferase assay

Luciferase activity was assayed 48 h after transfection using a Dual-Luciferase Reporter Assay System kit (Promega, China). Cells were washed with PBS(−) and harvested in 200 μL of Passive Lysis Buffer (PLB, Promega, China). After centrifugation at 12,000 rpm for 5 min at 4°C, the supernatant (100 μL) was transferred to a fresh 1.5 mL Eppendorf tube. Firefly and Renilla luciferase activity was measured in a Synergy Mx Monochromator-Based Multi-Mode Microplate Reader (BioTek, China). Firefly luciferase activity was measured for 10 sec following a 2 sec delay after the addition of the lysate (20 μL) to 100 μL of LAR II (Promega, China), and Renilla luciferase activity was measured for 10 sec following a 2 sec delay after the addition of 100 μL Stop & Glo® Reagent. Data are presented as the relative activity of Firefly luciferase to Renilla luciferase to normalize for transfection efficiency.

### Determination of α-galactosidase mRNA levels

Total RNA was extracted using Trizol reagent according to the manufacturer’s recommendations (Invitrogen, China). First-strand cDNA synthesis was performed using Oligo-(dT)_18_ (Invitrogen, China). The reaction mix was subjected to RT-PCR to detect levels of α-Gal and human β-actin mRNA. Human β-actin was used as an internal control. The relative mRNA levels were determined and analyzed using the ABI Prism 7000 Sequence Detection System (Applied Biosystems). Transfection and assessment experiments were repeated three times to analyze relative gene expression, and each sample was tested in triplicate.

### α-galactosidase activity assay

Enzyme activity was assayed in a reaction mixture containing 0.1 mL of McIlvaine buffer (Phosphate and citric acid ,100 mmol/L, pH = 4.8) and 0.5 mL of 20 mmol/L p-nitrophenyl-α-D-galactopyranoside (pNPG) substrate (den Herder et al., 1992) [24]. The reaction mixture was pre-incubated at 37°C for 5 min before adding 0.2 mL of enzyme solution (cell lysate). After 5 min, the reaction was stopped by adding 1.5 mL of 0.1 mol/L Na_2_CO_3_ solution. The released p-nitrophenol was determined spectrophotometrically at 405 nm using a calibration curve prepared with p-nitrophenol under the same conditions. One unit of α-galactosidase activity was defined as the amount of enzyme liberating 1 μmol p-nitrophenol in 1 min under the assay conditions. The amount of protein was determined using a Bio-Rad dye-binding assay (Bio-Rad Laboratories) and bovine serum albumin as a standard.

### Statistical analysis

All values are expressed as means ± SEM. The statistical significance of the differences among groups was analyzed by one-way ANOVA. Multiple comparisons among means were conducted using Tukey’s procedure when significant difference was identified. Difference was regarded as significant when P<0.05. All statistical analyses were performed using the SPSS 17.0 software package.

## Results

### Construction of reporter plasmids containing intestinal promoters: Comparative *In Vitro* study

**Figure 2:**
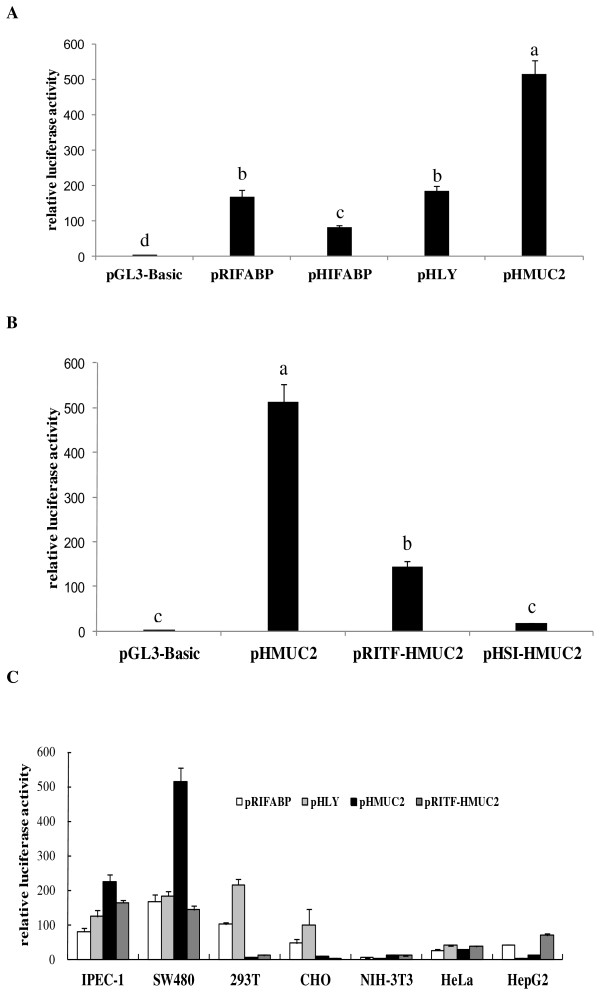
**Analysis of the activity and specificity of promoters**/**enhancers.** (**A**) Luciferase activity from the different intestine-specific promoters was analyzed by transient transfection in SW480 cells. (**B**) Influence of the HSI and RITF enhancer on human mucin-2 promoter (HMUC2) activity. (**C**) Luciferase activity of indicated plasmids in intestinal cells (SW480) and non-intestinal cells (CHO, HeLa, NIH-3T3, HepG2 and 293T). Data are presented as the relative activity of Firefly luciferase to Renilla luciferase in order to normalize transfection efficiency among different wells. The means ± SEM of luciferase expression driven from the selected intestine-specific promoter/enhancers are represented. Different letters represent significant difference, *P* < 0.01.

Figure 2A shows that the HMUC2 promoter has the highest activity among the intestine-specific promoters tested, which was about 2 to 3 times higher than that of the other three promoters (*P* < 0.05). RITF and HSI were unable to enhance the promoter activity of HMUC2, and surprisingly decreased (*P* < 0.05) the activity of HMUC2 3-fold and 30-fold, respectively (Figure 2B). Transfection of SW480, 293T, CHO, NIH-3T3, IPEC-1, HeLa and HepG2 cells showed that the HMUC2 promoter demonstrated better specificity in SW480 cells than the other promoters tested. In contrast, some intestine-specific promoters, such as HIFABP and HLY, could effectively drive luciferase expression in non-intestinal cells, especially in 293T and CHO cells (Figure 2C).

### Testing functional repressor binding to the lac operator

**Figure 3:**
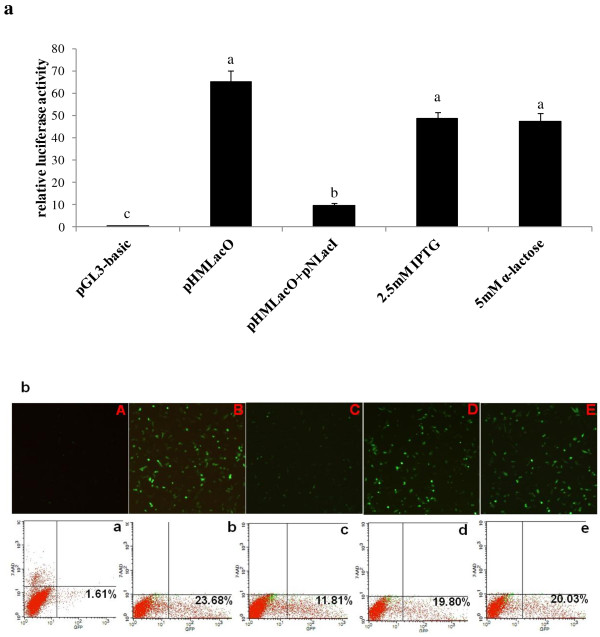
**Effects of IPTG and** α-**lactose on the transient expression of luciferase and green fluorescent protein** (**GFP**) **activity after transfection of SW480 cells with luciferase**-**containing and GFP**-**containing plasmids**, **respectively.** (**A**) Effects of inducers on the transient expression of luciferase activity; lysates were derived from SW480 cells transfected with luciferase-containing plasmids and incubated for 48 h before harvesting. Data are presented as relative activity of Firefly luciferase to Renilla luciferase in order to normalize transfection efficiency among different wells. Different letters represent significant difference, *P* < 0.05. (**B**) Qualitative analysis of inducer effect on transient expression of GFP activity. a–e: FACS analysis of cells transfected with vectors shown (A-E, A:pGL3-basic, B: pHMLacO, C: pNLacI and pHMLacO, D: inducer IPTG, E: inducer α-lactose). The percentage of cells exhibiting strong fluorescence (date marked area) was calculated as the number of cells exhibiting fluorescence.

Regulation of reporter gene expression by the modified *lac* operon system is shown in Figure 3. Co-transfection of pNLacI and pHMLacO yielded luc activity about 6.5-fold lower than with expression of pHMLacO alone (*P* < 0.05). When the inducers IPTG or α-lactose were added, luc activity was increased (*P* < 0.05) 5-fold and 4.8-fold, respectively, similar to that for transfection with pHMLacO alone. These results (Figure 3A) indicate that both IPTG and α-lactose could bind well to the repressor and therefore eliminate (*P* < 0.05) its inhibitory effect.

In addition, transient transfection with single pHMLacO-GFP resulted in the generation of cells with bright GFP-derived fluorescence (seen in Figure 3B). In contrast, co-transfection of pHMLacO-GFP and pNLacI gave rise to poor fluorescence. When the inducers (IPTG and α-lactose) were added, the fluorescence intensity was similar to cells transfected with pHMLacO-GFP alone. FACS analysis showed that the percentage of cells transfected with pHMLacO-GFP with GFP expression had a mean of 23.68%, while in SW480 cells transfected with pHMLacO-GFP and pNLacI, it was below 11.68%. The numbers of cells exhibiting high degrees of fluorescence increased to 19.80% and 20.03% when IPTG and α-lactose were added, respectively.

### Determination of α-galactosidase mRNA and protein levels

**Figure 4:**
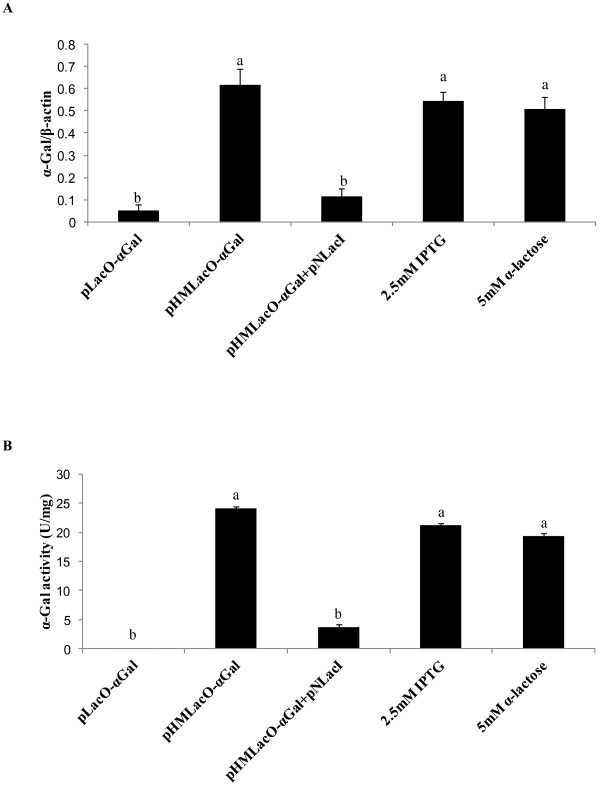
**Effects of inducers on the transient expression of α-galactosidase (α-Gal) mRNA and on α-Gal activity levels.** (**A**) RT-PCR analysis of α-Gal mRNA levels; β-actin was used to control transfection efficiency and cell recovery. (**B**) α-Gal activity assay. Values are the average of triple determinations with the SD indicated by error bars. Different letters represent significant difference, *P* < 0.01.

Both mRNA levels and enzyme activity of α-galactosidase were dramatically decreased (*P* < 0.05) by the introduction of a repressor. When the inducers IPTG and α-lactose were added, inhibition of the repressor was greatly relieved so that both the α-galactosidase mRNA levels and enzyme activity were increased (*P* < 0.05) by 5-fold and 7-fold, respectively (Figure 4).

## Discussion

Intestine-specific regulatory elements can achieve a maximal expression of candidate genes in the gastrointestinal gut. Previous work has shown that some intestine-specific promoters, such as HIFABP [9], RIFABP [10], HMUC2 [11] and HLY [12], can efficiently activate downstream gene expression in intestinal cells or tissues. However, the promoter conferring the highest activity has not yet been determined. Therefore, we first compared the transcriptional characteristics of these four promoters and found that the HMUC2 promoter had the highest activity in SW480 cells, which was about 2 to 3 times higher than that of the other three promoters. In addition, the HMUC2 promoter had low activity in NIH-3T3, 293T, CHO, HeLa and HepG2 cells. These results are similar to previous work by Gum et al. [11], who found that HMUC2 promoter activity was 4- to 6-fold higher in Cla cells than in other cell lines (L cells, AGS, HT-1080 and MGC80-3). Cla cells are a type of intestinal cell; however, HMUC2 promoter activity was not investigated in other typical intestinal cell lines, such as IPEC-1, SW480 or Coco-2. The activity of the HMUC2 promoter being highest in SW480 cells might relate to transcription factors, such as the SP1 family, CDX2 and GATA. Several lines of evidence demonstrate that MUC2 promoter activity is greatly reduced by mutation in the SP1 [25,26] and GATA-4 binding sites [27], while CDX2 binding to the MUC2 gene cis element results in a 5-fold enhancement in activity [28].

The other promoters we tested showed varying degrees of non-specific activity. Rottman and Gordon [29] reported that the RIFABP promoter has high activity in the Caco-2 cell line but still activates gene expression in HeLa and HepG2 cell lines, which was consistent with our results. Paneth cells of the small intestine are a rich source of lysozyme (encoded by *HLY*) [30], although the HLY promoter has low activity in the small intestine and can be detected in both HepG2 and U937 cell lines. In addition, *in vivo* experiments also support the conclusion that HLY promoter activity in the small intestine is only about 5% of that in the lungs in transgenic mice [12]. Hence, compared with IFABP and HLY promoters, the HMUC2 promoter might be the most appropriate regulatory element for intestine-specific gene expression.

To increase the strength of the intestine-specific promoters, we produced a series of chimeric sequences by combining the HMUC2 promoter with enhancers from the HSI and RITF genes. The HSI enhancer contains three nuclear protein-binding sites; SIF indicates the SI(sucrase-isomaltase enhancer) footprint ,stands for western blot. SIF1 is responsible for intestine-specific SI transcription and can act as an enhancer for the SI promoter [31]. SIF2 and SIF3 bind hepatocyte nuclear factor (HNF-1α and HNF-1β) [32] The RITF enhancer, containing a 9 base pair (CCCCTCCCC) element between −154 and −118 in the RITF promoter, is a cis-active element bound by a distinct nuclear transcription factor and is capable of increasing promoter activity [14]. We fused the RITF and HSI enhancers with the HMUC2 promoter, to generate a promoter predicted to have strong intestine-specific activity. However, the opposite results were observed; HMUC2 promoter activity was decreased 3-fold and 30-fold by the RITF and HSI enhancers, respectively. This result may be explained by promoter specificity and the location and orientation of a non-classical enhancer. Troelsen et al. [33] found that the intestinal lactase phlorizin hydrolase (LPH) enhancer is only active in front of intestine-specific promoters, such as LPH and SI promoters, but not the SV40 promoter. It is worth noting that the LPH enhancer could inhibit SI promoter activity irrespective of location and orientation. Thus, the effects of location and orientation of the HSI and RITF enhancers on the activity of intestine-specific promoters still needs further study.

Inducible systems that incorporate cell-specific promoters enable a target gene to be switched on and off repeatedly when needed without affecting the expression of non-targeted genes. In recent years, binary systems based on the interaction of two components, such as the *lac* operon system [15], the tetR-based system [16], the GAL4-based system [17] and the Cre/loxP recombination system [18] have been used to turn gene expression on and off. The VP16-activating domain in the TetR-based system and the GAL4-based system has been found to be toxic to cells [16]. The Cre/loxP system had been widely used in gene activation with non-reversible effects, while the *lac* operon system has successfully used mammalian regulatory elements to control gene expression in mammalian cells and transgenic mice for some time [15,19]. Most importantly, α-galactosidase gene expression based on the *lac* operon system could be increased along with the production of α-lactose because α-lactose is both the inducer of the *lac* operon system and the substrate of α-galactosidase. Therefore, we constructed a modified *lac* operon system consisting of two important components: the 116-bp operator and the repressor with a nuclear localization signal (NLS) added before the termination codon (TAG). In the *lac* operon system, luc activity and the percentage of cells with GFP expression were decreased 6.5-fold and 2-fold, and the α-galactosidase mRNA level and α-galactosidase activity were reduced 6-fold and 8-fold, respectively, due to inhibition by the repressor, which could be relieved when IPTG or α-lactose was added. These results were similar to those of previous studies [34].

We also found that the *lac* operon had a low-level leakiness; the target gene was still expressed at a low level when the repressor bound to the lac operator. This phenomenon was also investigated by Wyborski and DuCoeur, although they had successfully used the *lac* operon to regulate gene expression *in vivo*[20]. The *LacO* inserted at both −10 and −35 led to promoter activity being decreased 45-fold in the presence of a repressor [19]. In addition, the specificity of the repressor-operator interaction can be further increased by introducing a small degree of asymmetry in the operator; the symmetry in the operator alters the translational efficiency, but it cannot affect transcription [35]. We observed that α-galactosidase mRNA and protein levels have a similar trend, shown in Figure 4, which suggested that the location rather than the symmetry of the operator in the HMUC2 promoter should be changed to reduce the low-level leakiness. Re-encoding or mutating some amino acids in the *lacI* sequence can significantly improve suppression capability, as has been successfully shown by Mueller-Hartmann and Mueller-Hill [36]. Thus, the problem of low-level leakiness can be overcome in future work by altering the location of *LacO* and by introducing appropriate mutations into the *LacI* sequence.

α -galactosidase has drawn much attention due to its wide use in Fabry disease therapy and in feed additives to increase nutrition utilization efficiency and animal growth [4-6].

Although human α-galactosidase has been successfully applied in transgenic mice [1,7], it is uncertain whether bacterial α-galactosidase can be expressed in mammalian cells and transgenic animals. Some evidence reports that bacterial genes introduced into the mammalian genome are difficult to express due to methylation [37]. However, bacterial xylanase has been successfully used in transgenic mice, and activity of the enzyme can be detected up to 4.2 U/mg [38]. Our present results indicate that bacterial α-galactosidase can also be successfully expressed in mammalian cells and that α-galactosidase activity could be measured at about 24 U/mg. Several *in vivo* studies have shown that α-galactosidase supplementation with 0.08 U/kg feed in growing pig diets containing soybean meal and with 2.3 U/kg feed in piglet diets could significantly improve Gain:Feed by 6% and increase the digestibility of carbohydrates and protein in the small and large intestine [39,40]. Therefore, our inducible and highly-efficient α-galactosidase expression system might provide the basis for further transgenic pig production. The intestine-specific inducible system could also be beneficial for studying the function and effect of other exogenous genes in transgenic animals.

## Misc

Zhai Ya-Feng and Shu Gang contributed equally to this work.

## Competing interests

The authors declare that they have no competing interests.

## Authors' contributions

ZYF participated in the design of the study, carried out the experiments, statistical analysis and wrote the first draft of the manuscript. SG participated in the design of the study and the statistical analysis, and oversaw manuscript preparation. ZXT and ZZQ participated in the cell experiments and plasmid construction. WSB, WLN, ZYL and JQY participated in study design and coordination. LXJ participated in writing the final versions of the manuscript. All authors have read and approved the final manuscript.
